# Evaluation of Serum Levels of HER2, MMP-9, Nitric Oxide, and Total Antioxidant Capacity in Egyptian Breast Cancer Patients: Correlation with Clinico-Pathological Parameters

**DOI:** 10.3797/scipharm.1306-18

**Published:** 2013-09-22

**Authors:** Yara A. Rashad, Tawfik R. Elkhodary, Amal M. El-Gayar, Laila A. Eissa

**Affiliations:** 1 Department of Biochemistry, Faculty of Pharmacy, Mansoura University, Mansoura, 35516, Egypt.; 2 Oncology Center, Mansoura University, Mansoura, 35516, Egypt.

**Keywords:** Human epidermal growth factor receptor-2 (HER2), Matrix metalloproteinase-9 (MMP-9), Nitric oxide (NO), Total antioxidant capacity (TAC), Carcinoma Antigen 15-3 (CA15-3), Breast cancer

## Abstract

Breast cancer is by far the most common cancer in women worldwide and the main cause of cancer-related mortality. Breast cancer accounts for 38% of all malignancies among Egyptian women. The aim of our study was to evaluate the serum levels of human epidermal growth factor receptor-2 (HER2), matrix metalloproteinase-9 (MMP-9), nitric oxide (NO), and total antioxidant capacity (TAC) in breast cancer patients and to correlate these markers with clinico-pathological parameters. Serum HER2, MMP-9, and carcinoma antigen 15-3 (CA 15-3) were assessed in 80 breast cancer patients and ten healthy subjects as a control group by the enzyme-linked immunosorbent assay (ELISA) technique while NO and TAC were assessed by a colorimetric method. Serum HER2 was ≥15 ng/mL in nine patients (11.3%). High HER2 ECD levels were significantly associated with tissue HER2 (P<0.0001), metastasis (P= 0.0024), and negativity of both estrogen (P=0.0075) and progesterone (P=0.0239) receptors. Serum MMP-9 (P=0.0013), NO (P<0.0001), and CA 15-3 (P<0.0001) were significantly increased while serum TAC was significantly (P=0.01) decreased in breast cancer patients as compared to the control group. Serum MMP-9 was increased significantly (P=0.028) in metastatic patients as compared to non-metastatic patients. We found a positive correlation between serum HER2 and CA 15-3 (r=36, p=0.005). In conclusion, serum HER2 reflects the tissue HER2 status of breast cancer, so the determination of serum HER2 is helpful in assessing HER2 status, but in addition, a high level may reflect metastatic disease. Also, serum MMP-9 can be useful for denoting the development of metastasis in breast cancer patients. Follow-up is needed to evaluate the value of serum HER2 and MMP-9 as prognostic markers.

## Introduction

Breast cancer is by far the most common cancer in women worldwide and the main cause of cancer-related mortality [1]. In Egypt, as in many other parts of the world, breast cancer is the most common type of cancer: it accounts for approximately 38% of reported malignancies among Egyptian women [2].

The human epidermal growth factor receptor (HER)-2 (HER2/neu, c-erbB-2) is a transmembrane receptor that is overexpressed in 15–25% of breast cancers. HER2 overactivity is associated with adverse biological characteristics and poor clinical outcomes. Therefore, it is an attractive target for therapeutic intervention, a strategy that has been clinically validated [3]. HER2 is a member of the HER family of transmembrane receptors, which are characterized by the presence of an extracellular ligand-binding domain, an intracellular tyrosine kinase domain, and a cytoplasmic tail. HER2 can release its ECD through a proteolytic mechanism, known as ECD shedding [4]. Currently, tissue HER2/neu status has mainly been evaluated using immunohistochemistry (IHC) and fluorescent in situ hybridization (FISH) (when IHC results are equivocal) in the clinical setting [5]. The serum extracellular domain fragment of HER2 (HER2 ECD) represents a non-invasive and quantifiable biomarker that could supplement existing HER2/neu testing [6].

MMPs in humans are a family of 23 members that have been associated with remodeling of the extracellular matrix (ECM). Because of their involvement in the processing of ECM, MMPs were implicated in cancer invasion and metastasis [7]. HER2 shedding has been attributed to various zinc-containing metalloproteases including members of the ADAM (A Disintegrin And Metalloproteases) and MMP families [8]. On the basis of the detection of high activity of proMMP-9 in the sera and tissues of breast cancer patients, it is proposed that tissue MMPs may appear in the bloodstream in increased amounts in patients with biologically aggressive breast cancer, thereby providing a unique marker that might be useful for predicting the appearance of metastasis [9].

NO is a ubiquitous, water-soluble, free radical gas, which plays a key role in various physiological as well as pathological processes. It is synthesized by a complex family of nitric oxide synthase (NOS) enzymes [10]. Numerous clinical and experimental studies have implicated that NO and its derivatives have a critical impact on various carcinomas including breast cancer [11].

In order to develop an effective strategy to prevent and treat certain disorders, an accurate evaluation of ROS and antioxidants has a critical importance. The understanding of causes and progression of breast cancer contributes to the development of diagnosis and treatment. In addition to the parameters which are being used for following up cancer prevention, cancer diagnosis, and treatment, TAC level can also be used [12]. The major advantage of measuring TAC is to measure the antioxidant capacity of all antioxidants in a biological sample and not just the antioxidant capacity of a single compound.

## Results and Discussion

We measured serum HER2, MMP-9, NO, and TAC levels in patients with breast cancer and healthy controls and evaluated the value of these markers. The main tumor characteristics are summarized in Table 1. T1, T2 tumor size were reported in 48 patients (60%) and T3, T4 were reported in 32 patients (40%). Lymph node negative was reported in 15 patients (18.75%) and lymph node positive was reported in 55 patients (68.8%). Grade II was reported in 68 patients (85%) and grade III was reported in 12 patients (15%). Metastasis was present in 41 patients (51.25%) and absent in 39 patients (48.75%).

Receptor statuses of the patients are summarized in Table 2. Cancer tissue HER2 status was positive (3+ by immunohistochemistry [IHC] or 2+ by IHC with FISH amplification) in 20 patients (25.0%) and negative in 60 patients (75.0%).

Tumor therapy and surgical procedures of the breast cancer patients are summarized in Table 3. Breast conservative surgery was carried out in 2 patients (2.5%) and modified radical mastectomy in 67 patients (83.8%). Radiotherapy was delivered to 40 patients (50%). Chemotherapy was administered to 75 patients (93.6%). Hormonal therapy was administered to 43 patients (53.7%). In spite of trastuzumab approval for the treatment of HER2-positive patients, none of our patients in this study at that time received trastuzumab because of its high cost, but nowadays our policy is to administer trastuzumab to HER2-positive patients.

### Serum HER2 ECD

With a cut-off value of 15 ng/mL, 9 out of the 80 patients (11.3%) had HER2 ECD levels ≥15 ng/mL (high levels) and 71 out of the 80 patients (88.7%) had HER2 ECD levels <15 ng/mL (low levels).

### Association Between Tissue HER2 Status (IHC/FISH) and Serum HER2 ECD Levels

**Fig. 1:**
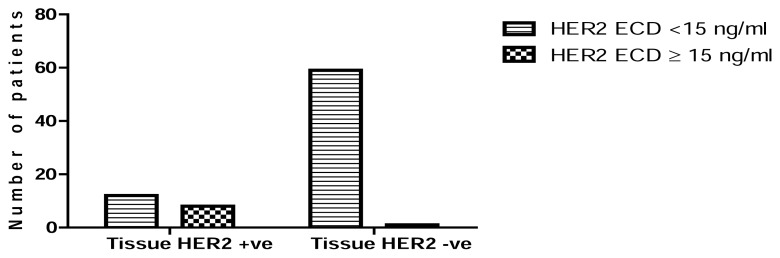
Association between tissue HER2 status and serum HER2 ECD levels.

The bivariate distributions of patients with tissue HER2 status and baseline serum HER2 ECD levels are shown in Table 4. High serum HER2 ECD levels were observed in 40% (8 out of 20) of patients with tissue HER2 positivity and in only 1.67% (1 out of 60) of the patients with tissue HER2 negativity. The P< 0.0001 indicated an association between high HER2 ECD levels and tissue HER2 status (Figure 1).

### Relationship between Serum HER2 ECD Levels and Clinico-Pathological Variables

**Fig. 2:**
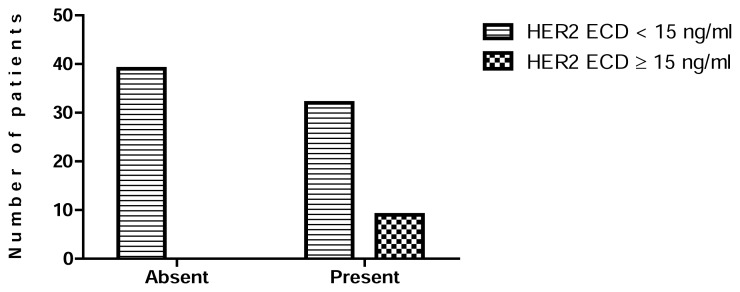
Association between metastasis and serum HER2 ECD levels.

**Fig. 3:**
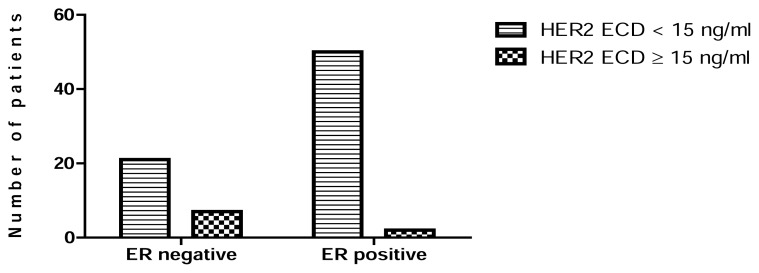
Association between ER receptor status and serum HER2 ECD levels.

**Fig. 4:**
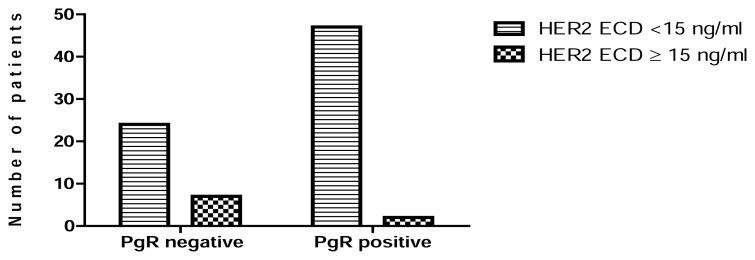
Association between PgR receptor status and serum HER2 ECD levels.

High HER2 ECD levels were significantly associated with metastasis (P = 0.0024) (Figure 2), negativity of ER (P = 0.0075) (Figure 3), and negativity of PgR (P = 0.0239) (Figure 4). No statistical relationship was found between HER2 ECD levels and the other variables, such as age, tumor size, and lymph node status (Table 5).

Determining the HER2/neu status is an integral part of the diagnostic work-up for breast cancer patients. The precise status of HER2 is clinically important because it directs the course of therapeutic treatments for breast cancer patients. The ECD of HER2 can be cleaved from the surface of breast cancer cells by MMPs and released into the serum, where it is detectable using an ELISA [13]. One original goal of serum HER2 ECD is to determine if this type of assay can be used to match the established FISH/IHC tests for HER2 status [6]. Serum HER2 could be used with tissue HER2 as a guide to trastuzumab therapy. The currently approved cutoff for an elevated serum HER2 is greater than 15 ng/mL, which results in a positive test in approximately 5% of healthy controls [14]. We also defined 15 ng/mL the upper limit of normal.

According to our study, an increased serum HER2 level was detected in approximately 11.3% of patients. This rate was close to the mean value of the worldwide studies published between 1992 and 2007 [15]. As shown in Table 4, the (P < 0.0001) indicated an association between high HER2 ECD levels and tissue HER2 status which agreed with Ludovini et al [16] and Tsai et al [17] (Figure 1). However, one of 60 patients was HER2 negative by IHC/FISH but positive by serum HER2 ECD. This patient was a locally advanced and metastatic patient, so this discrepancy may be related to the high tumor burden which is one of the factors associated with high serum HER2 ECD [16].

In our study, elevated levels of HER2 ECD were associated with the absence of ER (P=0.0075) and PgR (P=0.0239) (Table 5, Figures 3, 4). This result is in accordance with studies by (Ludovini et al [16], Tsai et al [17], Ratnatunga et al [18], and Ayadi et al [19]) who demonstrated an inverse association between hormone receptor expression and Her2 overexpression. Furthermore, a recent study has shown that HER family signaling through phosphatidylinositide 3-kinases (PI3-kinase) and serine/threonine-specific protein kinase (AKT) could decrease PR expression [20].

Elevated levels of HER2 ECD were associated with metastasis (P value= 0.0024) (Table 5, Figure 2) but no significant correlation between tissue HER2 status and metastasis was found (Table 6). Our results indicate that the serum level of HER2 ECD is important and has additional value in metastasis than in traditional IHC/FISH methods.

**Fig. 5:**
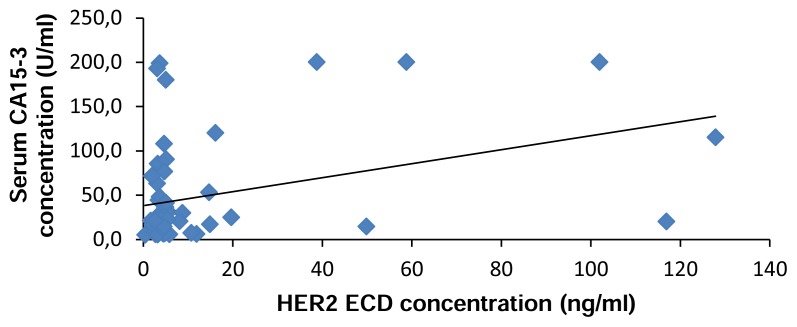
Significant positive correlation between HER2 ECD (ng/mL) and serum CA 15-3 (U/mL) concentration (r= 0.36, p= 0.005).

Serum HER2/neu results were correlated with serum CA 15-3 concentrations as continuous variables. There was a weak but significant correlation between the two markers, with a correlation coefficient of 0.36 (*P* = 0.005) (Figure 5). Similar results were confirmed by other studies like Molina et al [21] and Ali et al [22]. Of the 9 patients with increased serum HER2/neu, 5 (55.6%) also had increased CA 15-3 (more than 38.6 U/ml). Conversely, 14 of 71 (19.7%) of the patients who did not have increased serum HER2/neu had increased serum CA 15-3.

The serum levels of other studied parameters are summarized in Table 7.

CA 15-3 is the most widely used serum tumor marker for breast cancer. In the present study, serum CA 15-3 concentration was elevated significantly in breast cancer patients when compared to the healthy control group (P<0.0001) (Table 7), as reported by Atoum et al [23]. This result is expected because CA 15-3 was considered the most useful tumor marker for monitoring breast cancer patients.

MMP-9 has a causative role in cancer invasion and metastasis, and the development of MMP inhibitors as antimetastatic therapy has started some years ago [24]. As shown in Table 7, we observed significantly higher serum MMP-9 levels in the cancer patients when compared to the control (p= 0.0013). This result agreed with La Rocca et al [25] and Patel et al [26]. Moreover, the serum MMP-9 level was significantly higher in metastatic patients than non-metastatic patients (p= 0.028) (Table 8). This result agreed with Patel et al [26]. That indicated the important role of MMP-9 in the metastasis and invasion of breast cancer. Type IV collagen is abundant in basement membranes separating the epithelial cells from the underlying stroma. Increased expression and activity of MMP-9 in tumors leads to the degradation of basement membranes, an essential step in tumor invasion [27]. As shown in Table 8, no correlation was found between serum MMP-9 and other clinico-pathological parameters considered which agreed with Daniele et al [28].

**Fig. 6:**
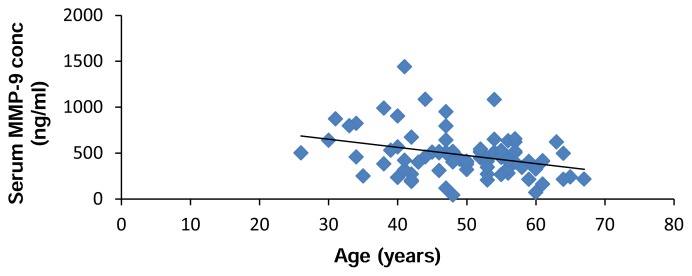
Significant negative correlation between serum MMP-9 concentration (ng/mL) and age (years) (r = −0.329, p = 0.003).

We found a negative correlation between serum MMP-9 and age (Figure 6).

We found no significant difference between HER2 ECD-positive (HER2 ECD ≥ 15 ng/mL) and -negative patients (HER2 ECD < 15 ng/mL) in terms of their serum MMP-9 (Table 9). This result agreed with Tsai et al [17]. This indicated that MMP-9 has no role in HER2 shedding, and suggested that another type of protease might be responsible for high serum HER2 ECD levels.

The role of NOS in tumor biology has not been defined clearly. It is said to have both tumoricidal as well as tumor-promoting effects which depend on its timing, location, and concentration [10]. In our study, breast cancer patients showed significant increases in serum NO levels when compared with control subjects (P<0.0001) (Table 7). However, there was no significant difference between metastatic and non-metastatic patients which agreed with Günel et al [29] and Konukoglu et al [30] (Table 8). These elevated NO levels might be a result of increased NOS II activity, which was stimulated by a host defense system against tumor growth. NO and NO-derived reactive nitrogen species induce oxidative and nitrosative stress which results in DNA damage (such as nitrosative deamination of nucleic acid bases, transition, and/or transversion of nucleic acids, alkylation, and DNA strand breakage) and the inhibition of DNA repair enzymes (such as alkyltransferase and DNA ligase) through direct or indirect mechanisms. The induction of mutations in cancer-related genes or post-translational modifications of proteins by nitration, nitrosation, phosphorylation, acetylation, or polyADP-ribosylation are some key events that can increase cancer risk [31]. No correlation was found between serum NO and the clinico-pathological parameters considered (Table 8).

One mechanism by which some physiological, physical, lifestyle, and nutritional factors may help to prevent cancer is through the modulation of oxidative stress status [32]. We observed significantly lower serum total antioxidant capacity (p= 0.01) levels in the cancer patients when compared to the control (Table 7). This result agreed with Sener et al [12]. No correlation was found between serum TAC and the clinico-pathological parameters considered (Table 8). In addition to the parameters which are being used for cancer prevention, diagnosis, and treatment, TAC can also be used. Decreasing of TAC may lead to breast cancer, so the antioxidants play an important role in the protection from breast cancer.

## Conclusion

Serum HER2 reflects the tissue HER2 status of breast cancer, so the determination of serum HER2 is helpful in the assessment of the HER2 status, but in addition a high level may reflect metastatic disease. Also, serum MMP-9 can be useful for denoting the development of metastasis in breast cancer patients. Follow-up is needed to evaluate the value of serum HER2 and MMP-9 as prognostic markers.

## Experimental

### Patients

Eighty female patients with breast cancer were recruited from the Oncology Center, Mansoura University, Mansoura, Egypt, diagnosed in the period from 2004 to 2012. Their ages ranged between 26–67 years with a mean ± SD of 49.58± 9. The study was approved by the local institutional ethical committee and patients’ consents were obtained according to the regulations of the Egyptian Ministry of Health. All cases involved in this study were clinically, radiologically, and pathologically examined in the Oncology Center, Mansoura University. Tumor staging followed the tumor–node–metastasis (TNM) American Joint Committee on Cancer classification [33]. Estrogen receptors (ERs), progesterone receptors (PgRs), and HER2 status were assessed at the time of surgery on formalin-fixed paraffin-embedded tissue blocks of the primary tumor in the Oncology Center. Recorded clinical and pathological features for each patient included age, menopausal status, histology, grade, ER and PgR status, stage, surgical treatment, and medical adjuvant therapy.

Patients with other types of malignancy, advanced organ failure, active infection, and advanced medical co-morbidity were excluded from the study.

### Control Group

A negative control group comprised 10 healthy females with no apparent evidence of active disease or a medical disorder. Their ages ranged between 39–53 years with a mean ± SD of 46.00 ± 4.47.

### Blood Sampling

Fasting blood samples were collected from all patients and control groups. This blood was left to clot for 20–30 min at room temperature, followed by centrifugation at 1500 g for 10 min. The serum was then transferred to a polypropylene tube and stored at −80°C until use.

### Measurement of Serum HER2 ECD

The HER2 ECD levels were measured using ELISA (eBioscience human sHER-2 platinum ELISA kit, San Diego, California, USA.), following the manufacturer’s recommended protocol. The concentration of HER2 ECD in the samples was determined by interpolation of the sample absorbance from the standard curve. The minimum detectable was 0.06 ng/mL. The calculated overall intra-assay coefficient of variation was 1.9% and the calculated overall inter-assay coefficient of variation was 5.8%. The upper limit of normal was defined as 15 ng/mL, as previously reported [34].

### Serum MMP-9 Assays

Serum MMP-9 (92 kDa Pro- and 82 kDa active forms) was determined using ELISA (R&D Systems, Catalog # PDMP900 ELISA kit, Inc., U.S.A.) as per the manufacturer’s protocol. The minimum detectable level of MMP-9 is typically less than 0.156 ng/mL.

### Serum Nitric Oxide Assays

NO concentrations were determined using (R&D Systems, Catalog # KGE001, Inc., U.S.A.). This kit determines NO concentrations based on the enzymatic conversion of nitrate to nitrite by nitrate reductase. The reaction is followed by colorimetric detection of nitrite as an azo dye product of the Griess Reaction.

### Serum Total Antioxidant Capacity Assays

The serum TAC was determined according to the method of Koracevic et al [35] by using a commercially available colorimetric kit (from Biodiagnostic, Giza, Egypt).

### Serum CA 15-3 Assays

Serum CA 15-3 was determined using an ELISA kit (MyBioSource, San Diego, California, USA).

### Detection of Metastasis

Diagnosis of metastasis was dependent on clinical examination including symptomatology, together with radiology including a plan X-ray, ultrasonography, CT, bone scan, and tumor marker (CA 15-3).

### Tissue ER and PgR Analysis

ER and PgR status were evaluated by IHC on formalin-fixed, paraffin-embedded tissue sections using monoclonal antibodies and a standard methodology. Slides were scanned microscopically using the Allred score on a scale of 0 to 8. A score greater than 2 (corresponding to as few as 1% to 10% weakly positive cells) was used to define ER and PgR positivity.

### Tissue HER2 Analysis

IHC staining of specimens was carried out on formalin-fixed, paraffin-embedded breast cancer tissues using the mAb CB11 which targets the intracellular domain of the HER2/neu protein (BioGenex, San Ramon, CA). According to the Hercep Test criteria, an immunoreaction was scored as: 3+ if >10% of tumor cells showed strong and complete membrane staining, 2+ if membrane positivity was moderate and complete in >10% cells, 1+ if membrane positivity was weak and incomplete in >10% cells, and 0 if membrane staining was absent or present in <10% cells. Tumors scored as 3+ were considered HER2-positive while tumors scored as 0/1+ were designated as HER2-negative. In 2+ tumors evaluated by IHC, FISH analysis was carried out, using the Abbott-Vysis Path Vysion HER2 DNA Probe Kit (Abbott Laboratories, Abbott Park, IL), following the manufacturer’s recommended protocol. The results were reported as the ratio between the average copy number of the HER2/neu gene and that of the chromosome 17 centromere, analyzing 60 neoplastic nuclei. Specimens with a signal ratio of <2.0 were considered as nonamplified and 2.0 or greater as amplified.

### Statistical Analysis

For descriptive statistics, the frequency and percentage were calculated for qualitative variables, the mean values ± standard deviation (SD) were used for quantitative variables. For comparison between the two groups Student’s t-test was used. For correlation, Pearson correlation was used. Statistical computations were done on a personal computer using the computer software SPSS version 18 (Chicago, IL, USA) and GraphPad Prism 5. The Fisher’s exact test was used to assess the association among the clinical–pathological features and the levels of HER2 ECD. All p-values were two-sided and a p-value of less than 0.05 was considered to indicate a statistically significant difference.

## Figures and Tables

**Tab. 1:** Tumor characteristics

	Breast cancer patients N (%)
Tumor Size	
T1	6 (7.5%)
T2	42 (52.5%)
T3	16 (20%)
T4	16 (20%)

Tumor Grade	
G II	68 (85%)
G III	12 (15%)

LN stage	
N0	15 (18.75%)
N1	20 (25.0%)
N2	16 (20.0%)
N3	19 (23.8%)
Unknown	10 (12.5%)

Metastasis	
Absent	39 (48.75%)
Present	41 (51.25%)

Tumor stage	
I	1 (1.25%)
II	15 (18.75%)
III	23 (28.75)
IV	41 (51.25%)

N: number of patients; LN: lymph node.

**Tab. 2:** Receptor status of the patients in our series

	Breast cancer patients N (%)
ER−ve	28 (35%)
ER+ve	52 (65%)

PgR−ve	31 (38.8%)
PgR+ve	49 (61.2%)

Tissue HER2−ve	60 (75.0%)
Tissue HER2+ve	20 (25.0%)

ER/HER2	
ER +ve/HER2 −ve	42 (52.5%)
ER +ve/HER2 +ve	10 (12.5%)
ER−ve/HER2+ve	10 (12.5%)

PgR+ve/ER+ve/HER2−ve	37 (46.25%)
PgR+ve/ER−ve/HER2−ve	2 (2.5%)
Triple negative	16 (20%)

N: number of patients; ER: estrogen receptor; PgR: progesterone receptor; HER2: human epidermal growth factor receptor-2.

**Tab. 3:** Surgical and cancer treatment

	Breast cancer patients (N=80)

	N %
Radiotherapy	
No	40 (50%)
RT	40 (50%)

Chemotherapy	
No	5 (6.2%)
FAC	51 (63.8%)
FAC-Tax	21 (26.2%)
CMF	1 (1.2%)
FAC-Xeloda	1 (1.2%)
Tax Gem	1 (1.2%)

Hormonal	
No	37 (46.2%)
TAM	33 (41.2%)
AI	8 (10.0%)
TAM-AI	2 (2.5%)

Surgery	
Not Done	11 (13.8%)
MRM	67 (83.8%)
BCS	2 (2.5%)

RT: Radiotherapy; FAC: 5fluorouracil Adriamycin cyclophosphamide; Tax: taxotere; CMF: cyclophosphamide methotrexate 5 fluorouracil; TAM: tamoxifen; AI: aromatase inhibitor; BCS: breast conservative surgery; MRM: modified radical mastectomy.

**Tab. 4:** Association between tissue HER2 status and serum HER2 ECD levels

	Tissue HER2 positive^a^	Tissue HER2 negative	Total	P value
Serum HER2 ECD				
High ≥15 ng/mL	8	1	9	p< 0.0001*
Low <15 ng/mL	12	59	71	
Total	20	60		

* significant at p<0.05;

a IHC 3+, IHC 2+ and FISH amplified; IHC: immunohistochemistry; FISH: fluorescence in situ hybridization; ECD: extracellular domain.

**Tab. 5:** Relationship between serum HER2 ECD levels and clinico-pathological variables.

Characteristics	HER2 ECD <15 ng/mL	HER2 ECD ≥15 ng/mL	P value

	N %	N %	
Age (year-old)			
≤ 50	38 (92.7%)	3 (7.3%)	0.306
> 50	33 (84.6%)	6 (15.4%)	
Metastasis			
Absent	39 (100%)	0 (0%)	0.0024*
Present	32 (78.1%)	9 (21.9%)	
Lymph node status			
Negative	15 (100%)	0 (0%)	0.329
Positive	49 (89.1%)	6 (10.9%)	
Tumor size			
Tumor size ≤3	25 (86.2%)	4 (13.8%)	0.716
Tumor size >3	46 (90.2%)	5 (9.8%)	
Receptor status (cut-off ≥ 10%)			
ER status			
ER negative	21 (75%)	7 (25%)	0.0075*
ER positive	50 (96.2%)	2 (3.8%)	
PgR status			
PgR negative	24 (77.4%)	7 (22.6%)	0.0239*
PgR positive	47 (95.9%)	2 (4.1%)	

* significant at p<0.05; ER: estrogen receptor; PgR: progesterone receptor.

**Tab. 6:** Association between tissue HER2 status and metastasis.

	Tissue HER2 positive^a^	Tissue HER2 negative	Total	P value
Metastasis				
Absent	9	30	39	0.798
Present	11	30	41	
Total	20	60		

a IHC 3+, IHC 2+ and FISH amplified; IHC: immunohistochemistry; FISH: fluorescence in situ hybridization; ECD: extracellular domain.

**Tab. 7:** Serum concentration of CA 15-3, MMP-9, nitric oxide, and total antioxidant capacity in breast cancer patients as compared to the control group (mean ± SD).

	Control group (N=10)	Breast cancer patients (N=80)	P value
CA 15-3 (U/mL)	4.92 ± 2.77	48.69 ± 57.94*	<.0001
MMP-9 (ng/mL)	305.86 ± 116.18	477.1 ± 243.08*	0.0013
Nitric oxide (μmol/L)	293.9 ± 54.64	440.8 ± 219.34*	<0.0001
TAC (mM/L)	1.29 ±.41	0.943 ±.39*	0.01

N: number of patients;

* Significant difference from control group at p < (0.05).

**Tab. 8:** Correlation between serum levels of study parameters and clinico-pathological characteristics of breast carcinoma (Mean ± SD).

Characteristics	MMP9 (ng/mL)	NO (umol/L)	TAC (mM/L)
ER			
ER negative	448.9 ± 242.5	447.7 ± 203.3	0.99 ±.4
ER positive	492.3 ± 244.4	437.1 ± 229.3	0.92 ±.4
PR			
PR negative	410.7 ± 237.8	453.7 ± 197.0	1.0 ±.4
PR positive	519.1 ± 239.3	432.7 ± 234.0	0.9 ±.4
Her2			
Her2 negative	462.0 ± 246.0	453.2 ± 232.1	0.9 ±.4
Her2 positive	522.4 ± 234.2	403.7 ± 175.7	1.0 ±.3
Tumor size			
T1–T2	496.7 ± 220.7	438.1 ± 195.0	0.9 ±.3
T3–T4	447.7 ± 274.3	444.8 ± 254.8	1.0 ±.5
Tumor grade			
GII	483.7 ± 248.3	428.2 ± 207.5	0.97±.4
GIII	439.7 ± 216.8	512.3 ± 276.9	0.79 ±.3
Lymph node stage			
Negative	411.1 ± 235.4	480.7 ± 220.8	1 ±.3
positive	468 ± 236.8	421.2 ± 218.8	0.93 ±.4
Metastasis			
Absent	416.3* ± 202.3	427.6 ± 202.5	0.94 ±.4
Present	534.9* ± 266.0	453.4 ± 236.0	0.95 ±.4

* significant at p<0.05.

**Tab. 9:** Relationship between Serum levels of MMP-9 and HER2 ECD (Mean ± SD).

	HER2 ECD <15ng/mL	HER2 ECD ≥15ng/mL	P value
MMP-9 conc (ng/mL)	462.9 ± 238.4	589.3± 265.4	0.14
